# *Neurobiology of Language*: Volume 4 Reviewers List

**DOI:** 10.1162/nol_e_00130

**Published:** 2023-12-14

Patti Adank

Georgios P. D. Argyropoulos

Kristijan Armeni

Christoph Aurnhammer

Nilgoun Bahar

Jana Basnakova

Laura Batterink

Idan Blank

Lindsay Bowman

Jonathan Brennan

Trevor Brothers

Adam Buchwald

Chiara Cantiani

Stefano Cappa

Micaela Chan

Luyao Chen

Yuchun Chen

Anna Chrabaszcz

Laurent Cohen

H. Branch Coslett

Jacqueline Cummine

Anila D’Mello

Ayoub Daliri

Nicola Del Maschio

Andrew Tesla DeMarco

Dirk Den Ouden

Michele T. Diaz

Anthony Steven Dick

Guosheng Ding

Nai Ding

Irene Echeverria-Altuna

Mark Eckert

Allyson Ettinger

Zohar Eviatar

Heather Flowers

Robert Frank

Stefan Frank

Jon Gauthier

Giulia Gennari

Fatemeh Geranmayeh

Laura Giglio

Adele Goldberg

Tamei Guo

Gesa Hartwigsen

Denise Y. Harvey

Andrea Helo

Alexander Huth

Maria Ivanova

Lisa Johnson

Ingrid Johnsrude

Sivan Jossinger

Iliana Karipidis

Carina Kauf

Yoon Kim

Jean-Rémi King

Swathi Kiran

Amanda LeBel

Minna Lehtonen

Hanjun Liu

Kep Kee Loh

James Magnuson

Elina Mainela-Arnold

Suhail Matar

Drew McLaughlin

Erin Meier

Katharina H. Menn

Lars Meyer

Angela Morgan

Riikka Mottonen

Caroline Nettekoven

Anni Nora

Sebastian Ocklenburg

Christophe Pallier

Francesca Peressotti

Franck Ramus

Jamie Reilly

Ariel Rokem

Rachel Romeo

William Schuler

Annemarie Seither-Preisler

Valerie L. Shafer

Cory Shain

Maria Silveri

Eleonore Smalle

Kate Stone

Simone Sulpizio

Dinglan Tang

Xing Tian

Rosario Tomasello

Bruce Tomblin

Guy Vingerhoets

Katharina Von Kriegstein

Xiaosha Wang

Janet F. Werker

Marko Wilke

Janina Wilmskoetter

Stephen M. Wilson

Ming Xiang

Maya Yablonski

